# Functional alterations of neutrophils from β-thalassemia patients exposed to *Pythium insidiosum*: An *ex vivo* study

**DOI:** 10.1371/journal.pone.0359846

**Published:** 2026-10-01

**Authors:** Pasinee Sangsiwarit, Wisitsak Phoksawat, Nattiya Teawtrakul, Sonwit Phanabamrung, Parama Budmala, Ariya Chindamporn, Pratsanee Hiengrach

**Affiliations:** 1 Department of Microbiology, Faculty of Medicine, Khon Kaen University, Khon Kaen, Thailand; 2 Division of Hematology, Department of Internal Medicine, Faculty of Medicine, Srinagarind Hospital, Khon Kaen University, Khon Kaen, Thailand; 3 Department of Microbiology, Faculty of Medicine, Chulalongkorn University, Bangkok, Thailand; 4 Research and Diagnostic Center for Emerging Infectious Diseases (RCEID), Faculty of Medicine, Khon Kaen University, Khon Kaen, Thailand; Ramathibodi Hospital, Mahidol University, THAILAND

## Abstract

Patients with β-thalassemia are highly susceptible to *Pythium insidiosum*, the causative agent of human pythiosis, a severe and often life-threatening disease in tropical regions. This vulnerability has been associated with iron overload and immune dysregulation. However, the underlying mechanisms of impaired host defense, especially in neutrophils, remain incompletely understood. This study investigated neutrophil function in response to *P. insidiosum* zoospores, using neutrophils isolated from β-thalassemia patients and comparing their responses with those from healthy controls. *Ex vivo* study, isolated neutrophils were co-incubated with *P. insidiosum* zoospores, then evaluated for pathogen-killing activity, neutrophil extracellular trap (NET) formation, and phagocytic capacity. Neutrophils isolated from β-thalassemia patients exhibited significantly reduced fungicidal activity, as indicated by higher fungal burdens in comparison with healthy controls. In addition, NET formation was significantly diminished in the neutrophils isolated from the patient group, reflecting impaired extracellular pathogen containment. Similarly, phagocytic activity in the neutrophils isolated from the healthy group was significantly higher than in the patient group. These findings demonstrate that impairments of neutrophil functions, including killing activity, NET formation, and phagocytic capacity, contributed mechanistically to the increased susceptibility to *P. insidiosum* in patients with β-thalassemia. This study highlights the importance of targeting innate immune dysfunction in the development of therapeutic strategies for human pythiosis.

## Introduction

Pythiosis is a life-threatening infectious disease caused by *Pythium insidiosum*, an organism classified within the phylum Oomycota of the kingdom Chromista [1], distinct from true fungi because it produces motile, biflagellate zoospores [2]. *Pythium* zoospores facilitate infection by actively swimming in stagnant aquatic environments, which are prevalent in tropical regions, especially Thailand [3,4], to colonize exposed host tissues. High mortality rates have characterized pythiosis, and it is frequently misdiagnosed [5], as its clinical presentation mimics fungal infections but does not respond to conventional antifungal therapies. Previous reports suggest that a critical risk factor for pythiosis is thalassemia, especially β-thalassemia [6,7].

β-thalassemia is a hereditary blood disorder characterized by deficient or absent production of β-globin chains, which are essential components of hemoglobin [8]. This genetic impairment typically manifests as microcytic anemia. A primary consequence of this condition is the development of systemic iron overload [9], which triggers oxidative stress and cellular injury, directly contributing to clinical complications such as immune abnormalities and increased vulnerability to severe infections [10]. Within this context, neutrophils serve as the critical first line of the innate immune system. They are responsible for capturing and eliminating pathogens during the initial stages of fungal infection, acting as a vital defense before the activation of more complex humoral and lymphocyte-mediated responses. Neutrophils employ a combination of phagocytosis, reactive oxygen species (ROS) production, degranulation, and the formation of neutrophil extracellular traps (NETs) to control the pathogen [11]. Previous reports suggest that the filamentous hyphae of *P. insidiosum* are often too large for ingestion, thereby necessitating reliance on extracellular mechanisms [12].

While the baseline activity of neutrophils against *P. insidiosum* is documented, their specific behavior within the β-thalassemia population has not been fully elucidated. We aim to assess the functional response of neutrophils isolated from β-thalassemia patients following exposure to *P. insidiosum* zoospores and compare them with those of neutrophils isolated from healthy individuals in an *ex vivo* study. Then, particular focus on phagocytic capacity, fungicidal activity, and NET formation, all of which are critical for controlling filamentous pathogens. Therefore, this study is the first *ex vivo* study to investigate the mechanisms underlying neutrophil dysfunction against *P. insidiosum* in patients with β-thalassemia. Additionally, this knowledge is essential for improving clinical outcomes.

## Methods

### Subject selection

All eligible participants aged between 18 and 60 years were included. Blood samples were collected from 12 donors, six with β-thalassemia major and six healthy controls. The recruitment period was from 21^st^ December 2025–29 ^th^ May 2026. The diagnosis of β-thalassemia major was established based on clinical history, hematological parameters, and hemoglobin electrophoresis, in accordance with standard protocols employed at the Hematology Clinic, Srinagarind Hospital, Khon Kaen, Thailand. Following the obtainment of written informed consent, blood samples were collected via phlebotomy in accordance with the protocol approved by the Center for Ethics in Human Research at Khon Kaen University (HE681691).

### Inclusion criteria

Inclusion criteria for the patient group required hemoglobin (Hb) levels less than 9 g/dL and serum ferritin levels higher than 1,000 g/dL. Conversely, individuals with coexisting autoimmune diseases or active infections were excluded from the study. The control group comprised healthy individuals aged 18–60 years with no history of blood disorders or chronic illnesses affecting iron metabolism [13]. Participants were required to have Hb levels between 12 and 18 g/dL [14] and serum ferritin levels within the normal range between 15 and 300 μg/L [15].

### Exclusion criteria

Any individual who was unable to provide informed consent or who had illnesses or treatments that might affect the biomarkers under investigation was excluded. Furthermore, women who were breastfeeding or pregnant were not allowed.

### Neutrophil isolation

Heparinized blood from six patients with β-thalassemia and six healthy donors was recruited from Srinagarind Hospital, Khon Kaen, Thailand. Human neutrophils were isolated from whole blood using a standard density gradient separation method, as previously described [12]. Briefly, fresh heparinized blood was layered onto Polymorphprep™ (Axis-Shield, Norway) and centrifuged at 1,800 rpm for 30 min at 25°C, resulting in the separation of six distinct bands, including plasma, monocytes, neutrophils, isolation media, and a red blood cell pellet. Residual red blood cell contamination was eliminated using a red blood cell lysis buffer (Gibco-Invitrogen, USA). The resulting neutrophil pellet was resuspended in RPMI 1640 medium (Gibco-Invitrogen) supplemented with 10% heat-inactivated fetal bovine serum (FBS; Gibco-Invitrogen). Cell viability and purity were assessed via trypan blue (Sigma-Aldrich, USA) exclusion and Wright-Giemsa staining, respectively. To maintain viability, neutrophils were rested in complete RPMI 1640 at 37°C in a 5% CO_2_ incubator before experimental use. However, because neutrophils have a short lifespan, all separation procedures were initiated within 2–3 h of blood collection.

### Cultivation of *P. insidiosum* and induction of zoospores

A clinical strain of *P. insidiosum*, previously isolated from a patient with vascular pythiosis, was cultured in Sabouraud dextrose broth (SDB; Oxoid, UK) overnight. Zoospore production was performed as described by Mendoza and Prendas [16]. Briefly, the microorganism was cultured on sterile blanket grass (Axonopus compressus) on malt agar (Oxoid) at 37°C for 48 h. To induce the formation of encysted zoospores, the blanket grass colonized with *Pythium* hyphae was transferred to an induction medium containing calcium (Ca^2+^) and magnesium (Mg^2+^) ions, which facilitate *Pythium* zoospore production [17]. Finally, the encysted zoospores were washed with 1X phosphate-buffered saline (PBS, pH 7.4) and quantified via trypan blue before use.

### Measurements of neutrophil-associated parameters

#### Evaluation of the fungicidal activity of neutrophils.

To specifically evaluate the killing capacity of neutrophils derived from β-thalassemia patients against *P. insidiosum* infection compared to healthy donors, isolated neutrophils were co-incubated with live *Pythium* zoospores in 24-well plates using complete RPMI 1640 medium supplemented with 10% heat-inactivated FBS. This co-culture was maintained at a multiplicity of infection (MOI) of 1 for durations of 0 and 60 min. Following incubation, 100 µL of the challenged samples were spread onto blood agar (Oxoid) and incubated overnight at 37°C, followed by manual colony enumeration.

#### Assessment of NET formation.

To evaluate the interaction between *P. insidiosum* zoospores and NET formation, the experiment was performed as described in the previous publication [12]. Zoospore isolates were heat-treated at 80°C for 30 min with gentle agitation, then centrifuged at 5,000 rpm for 10 min to harvest the cell pellet. Neutrophils isolated from healthy donors (control) and β-thalassemia patients were seeded onto poly-L-lysine-coated coverslips (Sigma-Aldrich) at a density of 10^5^ cells. The cells were subsequently challenged with 100 ng/mL of 12-myristate 13-acetate (PMA; Sigma-Aldrich) as a positive control, or heat-killed *Pythium* zoospores were added at an MOI of 1 and incubated for 2 h at 37°C in a 5% CO_2_ atmosphere. Coverslips were washed with Tris-buffered saline (TBS; pH 7.6) containing 0.05% Tween 20 and fixed with 4% (v/v) paraformaldehyde for 10 min. The samples were permeabilized with 0.1% Triton X-100 for 1 min at room temperature, blocked with 3% (w/v) bovine serum albumin (BSA; Sigma-Aldrich) supplemented with 5% FBS in 1X TBS for 30 min, and subjected to three washes with 1X TBS containing 0.05% Tween 20. Neutrophil degranulation and NET structures were visualized using antibodies against myeloperoxidase (MPO; BioLegend, USA) and neutrophil elastase (NE; BioLegend), while nuclear morphology was counterstained with 4′,6-diamidino-2-phenylindole (DAPI; Sigma-Aldrich). Fluorescent images were captured using fluorescence microscopy and independently evaluated by two blinded observers at 200 × magnification across 10 randomly selected fields.

#### Evaluation of cellular phagocytic activity by flow cytometric analysis.

Phagocytic activity was assessed using pHrodo-succinimidyl ester (Invitrogen, USA), which emits red fluorescence upon internalization into acidic phagosomes. Heat-killed *Pythium* zoospore preparation was prepared following a previously established protocol [18]. Briefly, the heat-killed zoospores were incubated with diluted pHrodo for 30 min, then washed three times with 1X PBS (pH 7.4). To evaluate phagocytosis, stained *Pythium* zoospores were co-incubated with isolated neutrophils from either healthy individuals or β-thalassemia patients at 37°C in a 5% CO_2_ atmosphere for 30 min. The cold co-incubation at 4°C was used as a negative control following the previous publication [19]. Following incubation, samples were blocked with FACS buffer supplemented with human AB serum at 4°C for 20 min. Neutrophils were then labeled with 0.2 mg/mL of anti-CD11b antibody (APC; BioLegend). Flow cytometric analysis was performed using a BD FACSLyrics (BD Biosciences, USA). Phagocytosed zoospores within neutrophils were detected in the PE channel (575 nm), and the presence of PE-positive cells served as an indicator of phagocytic uptake.

### Statistical analysis

Statistical analyses were performed using GraphPad Prism 8 (GraphPad Software, San Diego, CA, USA). The normality of data distribution was first evaluated to determine the appropriate statistical tests. For normally distributed data, one-way analysis of variance (ANOVA) was employed as appropriate, followed by suitable post hoc tests for multiple comparisons. Statistical significance was defined as a *p-value < 0.05*.

## Results

### Reduced fungicidal capacity of neutrophils isolated from β-thalassemia patients

Serum ferritin levels, an important parameter for disease monitoring, were measured in all participants. The results showed Hb and serum ferritin levels in comparison between healthy controls and patients with β-thalassemia (mean ± SD) (Table 1).

**Table 1:** Clinical characteristics of healthy donors and β-thalassemia patients.

Characteristics	Healthy donors (*n = 6*)	β-thalassemia patients (*n=6*)
Age at enrollment, years, mean ± SD	26.83 ± 3.37	40.5 ± 10.00
Sex, *n* (%)		
male	2 (33.3)	2 (33.3)
female	4 (66.7)	4 (66.7)
Hb (g/dL), mean ± SD	13.35 ± 1.58	7.52 ± 0.95
Serum ferritin (μg/mL), mean ± SD	98.80 ± 87.71	3,242.83 ± 2,384.78

Due to their involvement in host defense against *P. insidiosum* infection, the fungicidal activity of neutrophils was evaluated. The results demonstrated that after 60 min of co-incubation (MOI 1), neutrophils isolated from healthy donors significantly reduced the viability of *P. insidiosum* (124,388.889 ± 20,963.264) compared with baseline (0 min) (194,333.333 ± 9,679.842, *p = 0.0058*), indicating robust antimicrobial activity. In contrast, neutrophils isolated from patients with β-thalassemia exhibited a progressive increase in fungal burden at 60 min (259,111.111 ± 17,157.893) compared to 0 min (177,500.000 ± 17443.888, *p = 0.0005*). Furthermore, at 60 min, the fungal colony in the β-thalassemia group was significantly higher than in healthy controls (*p < 0.0001*). This marked elevation in fungal burden underscores a profound impairment in antimicrobial effector function within the patient cohort compared to the healthy control group (Fig 1).

**Fig 1:**
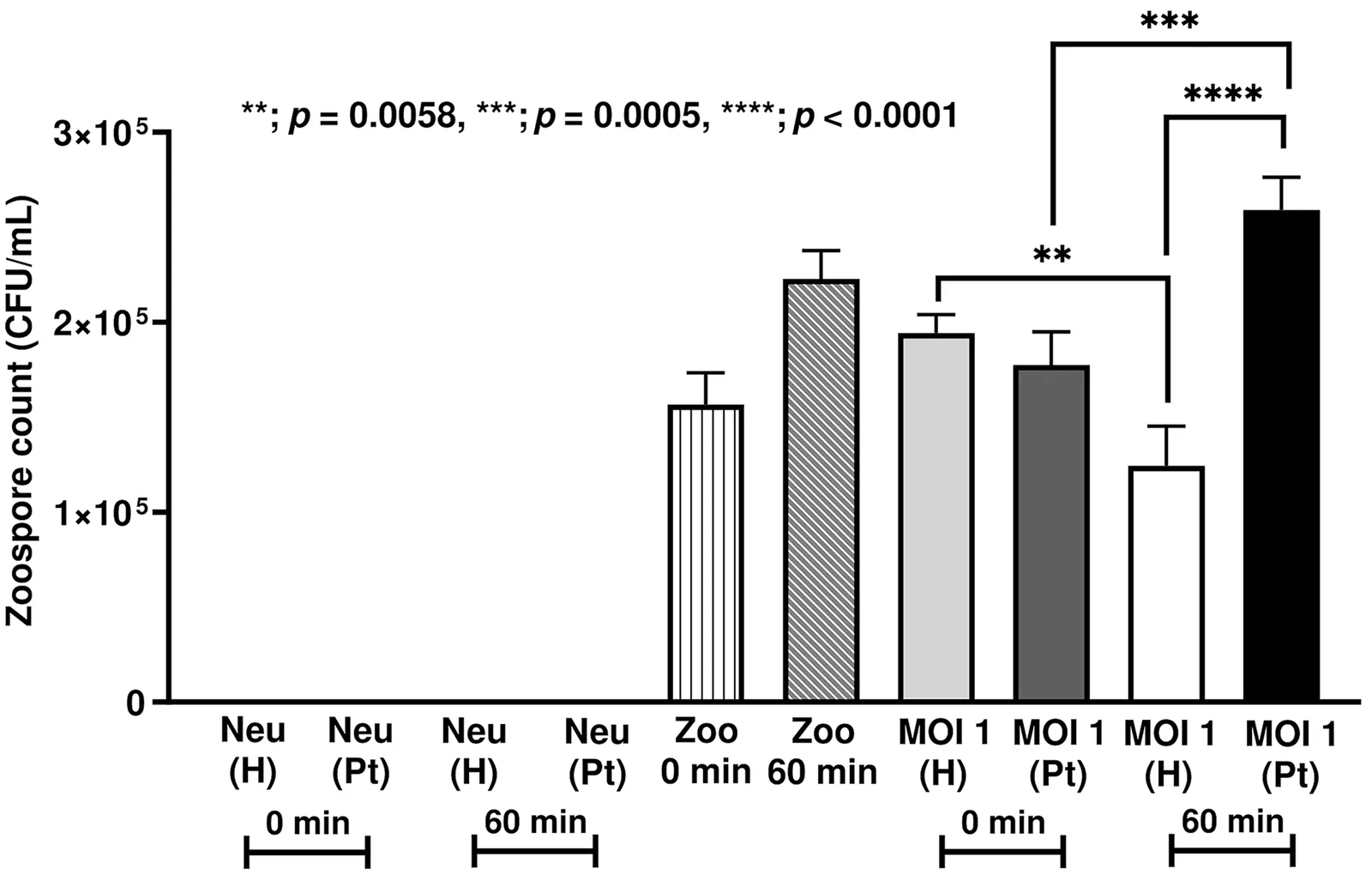
The characteristics of isolated neutrophils after co-incubation with zoospores of *P. insidiosum* are demonstrated based on killing activity analysis. Data were collected in triplicate for each participant. The data are shown as the mean ± S.E.M. (n = 6/group), ****, *p* < 0.0001 MOI 1 of β-thalassemia patients vs. MOI 1 of healthy controls; **, *p* = 0.0058 MOI 1 of healthy controls at 0 min vs. MOI 1 of healthy controls at 60 min; ***, *p* = 0.0005 MOI 1 of β-thalassemia patients at 0 min vs. MOI 1 of β-thalassemia at 60 min, using One-way ANOVA analysis. Abbreviations are as follows: Neu (H), neutrophils isolated from healthy individuals; Neu (Pt), neutrophils isolated from β-thalassemia patients; Zoo, *P. insidiosum* zoospores; MOI 1 (H), co-incubation of neutrophils isolated from healthy individuals with *P. insidiosum* zoospores at a multiplicity of infection (MOI) of 1; MOI 1 (Pt), co-incubation of neutrophils isolated from β-thalassemia patients with *P. insidiosum* zoospores at MOI of 1.

### Reduced NET formation of neutrophils isolated from β-thalassemia patients

The capacity of neutrophils to capture and inhibit *P. insidiosum* was assessed, as NET formation represents a critical host defense mechanism against large particles and invading pathogens. NET-associated markers, including MPO and NE, were evaluated. The results demonstrated that unstimulated neutrophils isolated from both healthy donors (Fig 2A) and β-thalassemia patients (Fig 2B), serving as negative controls, exhibited minimal baseline production of these markers, with no statistically significant difference between the two groups. Upon stimulation with either PMA, used as a positive control, or *P. insidiosum* zoospores, the release of MPO and NE significantly increased in both groups compared with unstimulated conditions (*p < 0.0001*). This result confirms that neutrophils remain responsive to both chemical and pathogen-derived stimuli regardless of the health status of donors (Fig 2A-E).

**Fig 2:**
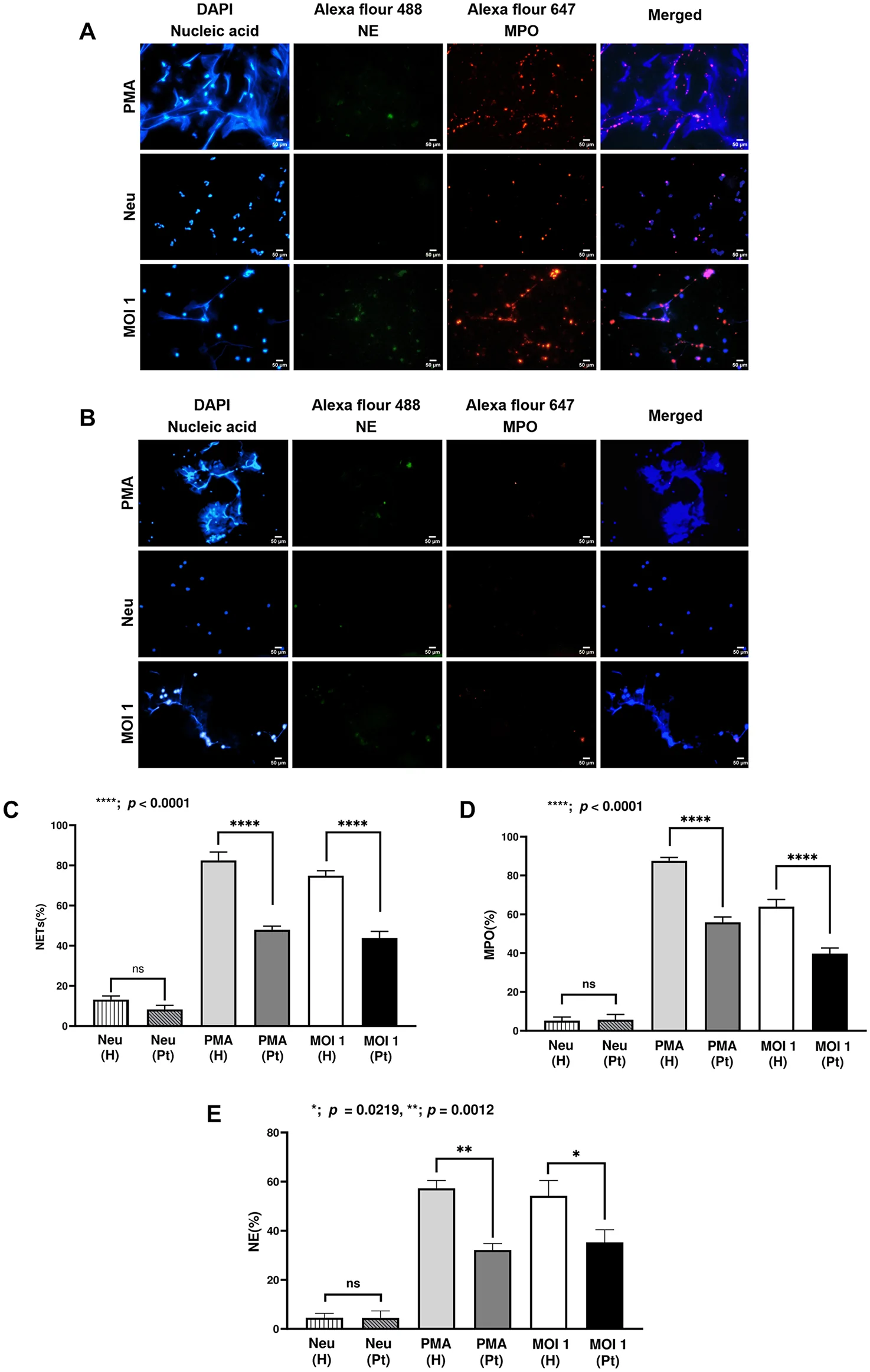
The characteristics of NET formation captured by a fluorescence microscope (original magnification, 200 × ; scale bars = 50 μm) showing neutrophils co-cultured with PMA (100 ng/mL; positive control) or *Pythium* zoospores. The nucleus was stained with DAPI (blue), neutrophil elastase (NE; green), and myeloperoxidase (MPO; red), with merged images used for identification of NETs of healthy donors (A) and β-thalassemia patients (B). The percentage of NET-releasing neutrophils was quantified relative to the negative control (unstimulated neutrophils) (C), which was expressed as the number of MPO-positive cells (D) and NE-positive cells (E) per 100 neutrophils. The data are shown as the mean ± S.E.M., ****, *p-value <* 0.0001; **, p-value = 0.0012; *, p-value = 0.0219; ns, p-value > 0.0001 MOI 1 of β-thalassemia patients vs. MOI 1 of healthy controls, using One-way ANOVA analysis (n = 6/group). Abbreviations are as follows: NE, neutrophil elastase; MPO, myeloperoxidase; NET, neutrophil extracellular trap; DAPI, 4′,6-diamidino-2-phenylindole; PMA, 12-myristate 13-acetate; Neu, neutrophils; Neu (H), neutrophils isolated from healthy individuals; Neu (Pt), neutrophils isolated from β-thalassemia patients; MOI 1 (H), co-incubation of neutrophils isolated from healthy individuals with *P. insidiosum* zoospores at a multiplicity of infection (MOI) of 1; MOI 1 (Pt), co-incubation of neutrophils isolated from β-thalassemia patients with *P. insidiosum* zoospores at MOI of 1.

Additionally, quantitative analysis revealed that neutrophils isolated from β-thalassemia patients produced significantly lower NET formation levels (Fig 2C), as assessed by MPO staining, compared with those from healthy individuals in both PMA and *P. insidiosum* stimulation. In brief, PMA-stimulated NET formation was significantly reduced in neutrophils isolated from the patient group (55.907 ± 2.703) compared with healthy controls (87.570 ± 1.738; *p* < 0.0001) (Fig 2D). Similarly, upon *P. insidiosum* zoospore stimulation, the neutrophils isolated from β-thalassemia generated lower levels of NETs (39.787 ± 2.869) than healthy neutrophils (63.912 ± 3.754; *p* < 0.0001). Consistently, NE release was significantly reduced in β-thalassemia neutrophils compared with healthy controls following both PMA and *P. insidiosum* stimulation (Fig 2D). In short, PMA-stimulated neutrophils isolated from β-thalassemia patients released lower levels of NE (32.165 ± 2.594) than those from healthy donors (57.314 ± 3.194; *p* = 0.0012). Likewise, *P. insidiosum*-stimulated neutrophils exhibited significantly reduced NE release in the patient group (35.278 ± 5.093) compared with healthy controls (54.223 ± 6.209; *p* = 0.0219) (Fig 2E). These findings suggest an impaired NETotic capacity in the β-thalassemia patients, which may compromise the extracellular antimicrobial defense against *P. insidiosum*.

### Reduced phagocytic activity of neutrophils isolated from β-thalassemia patients

The observed decrease in *Pythium* colony counts suggests a robust neutrophil-mediated defense. Therefore, the phagocytic capacity of neutrophils isolated from β-thalassemia patients against *P. insidiosum* was investigated. Neutrophils isolated from both healthy controls and β-thalassemia patients were able to phagocytose heat-killed *P. insidiosum* zoospores, compared with neutrophils alone and a negative control (co-incubation at 4°C). The quantitative analysis revealed that the phagocytic activity of neutrophils isolated from β-thalassemia patients was significantly lower than that of healthy controls (1.193 ± 0.276 vs. 0.120 ± 0.056, *p* = 0.0335). These findings indicate that the phagocytic capacity of β-thalassemia neutrophils to engulf *P. insidiosum* zoospores was impaired (Fig 3A–C).

**Fig 3:**
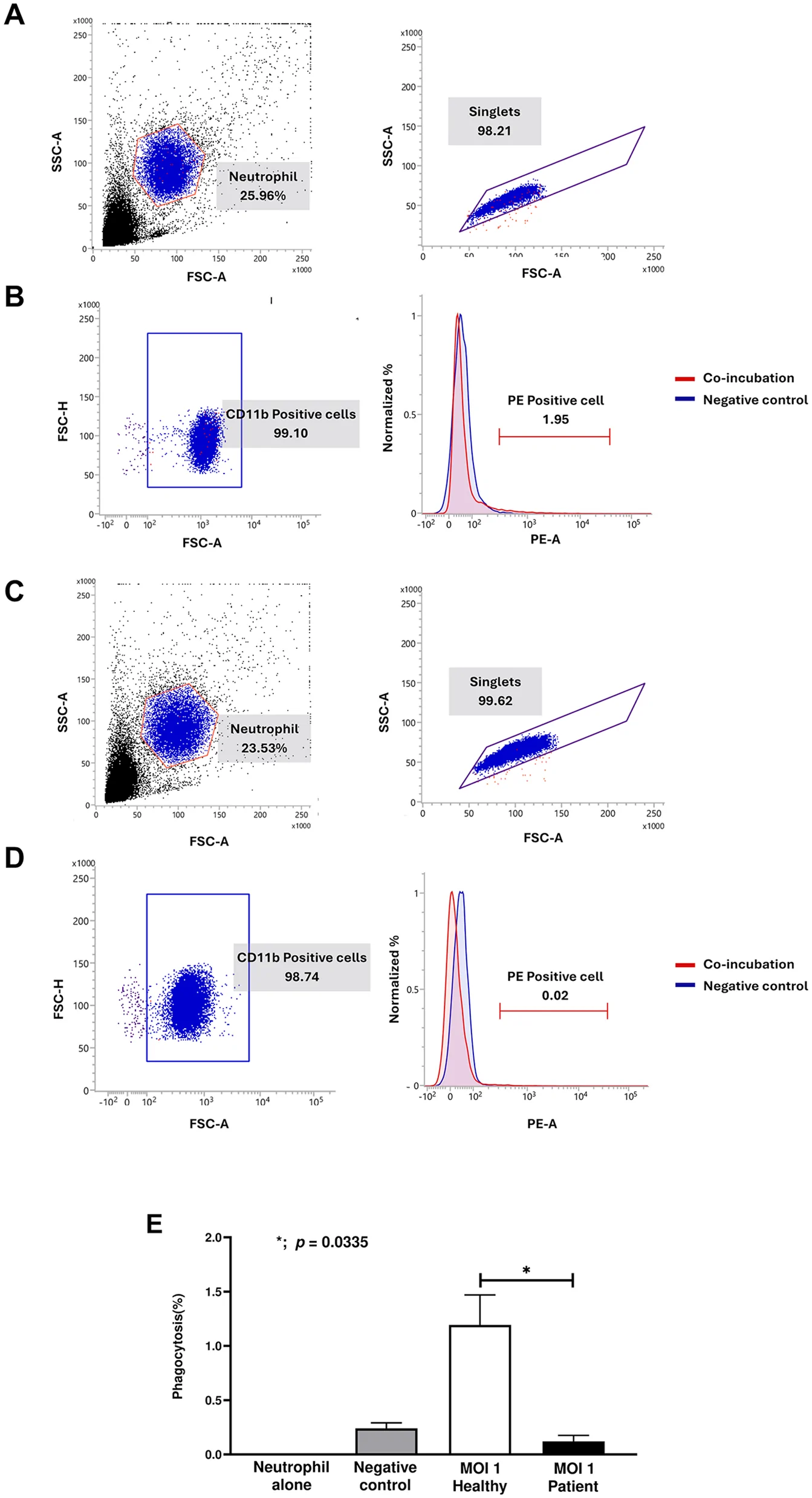
Representative flow cytometric analysis of phagocytic capacity in neutrophils following co-incubation with zoospores, showing flow cytometry plots and histograms of gated neutrophils from healthy donors (A, B). Representative flow cytometry plots and histograms of gated neutrophils from β-thalassemia patients (C, D). PE-positive cells indicate neutrophils that had phagocytosed *P. insidiosum* zoospores. Neutrophils co-incubated with zoospores at 4°C were used as a negative control. Quantification of the percentage of neutrophil phagocytosis after incubation with zoospores, compared with negative controls (E). The data are shown as the mean ± S.E.M., *, *p-value* = 0.0335 vs. MOI 1 of β-thalassemia patients, using the Kruskal-Wallis test (n = 6/group). Abbreviations are as follows: MOI 1, co-incubation of neutrophils isolated from either healthy individuals or β-thalassemia patients with *P. insidiosum* zoospores at a multiplicity of infection (MOI) of 1.

## Discussion

Pythiosis is a life-threatening infectious disease affecting both humans and animals [5,20], predominantly occurring in tropical, subtropical, and some temperate regions worldwide, with a particularly high incidence reported in Thailand [3,21]. The disease is caused by *P. insidiosum*, an aquatic oomycete pathogen that grows in swampy environments and stagnant water [16,22]. In humans, pythiosis is clinically classified into four major forms based on the anatomical site of infection and disease severity, including vascular pythiosis, ocular pythiosis, cutaneous pythiosis, and disseminated pythiosis [3]. Interestingly, vascular pythiosis is a severe clinical form of pythiosis, characterized by arterial invasion, thrombosis, and limb ischemia, often requiring surgical intervention and associated with substantial morbidity and mortality [3,21,23]. Notably, vascular pythiosis has been strongly linked to underlying hematological disorders, particularly β-thalassemia [24]. Several studies have reported a high prevalence of β-thalassemia among patients with vascular pythiosis, suggesting a disease-specific susceptibility in this population [6,24,25]. The pathophysiological basis for this association is not yet fully understood; however, it is thought to involve immune dysregulation, chronic hemolysis, and iron overload, all of which may compromise host defense mechanisms [26], especially neutrophil functions [27]. In particular, impaired neutrophil function and altered innate immune responses have been proposed as contributing factors that increase vulnerability to *P. insidiosum* infection in β-thalassemia patients.

This study demonstrates that neutrophils isolated from patients with β-thalassemia exhibit a significant reduction in fungicidal activity against *P. insidiosum* compared with healthy controls. The resulting increase in fungal burden reflects an inefficiency in pathogen clearance, whereas neutrophils from healthy individuals effectively restricted *P. insidiosum* growth, indicating preserved effector function. Interestingly, the results demonstrated that after 60 min of incubation, *Pythium* zoospores co-incubated with neutrophils isolated from β-thalassemia exhibited a trend toward higher colony counts compared with the zoospore alone (control group). This might be associated with both neutrophil dysfunction in this group of patients [10] and the dual role in iron management by controlling the organism and, ironically, releasing iron into the surrounding environment as a nutrient source for microorganisms [28], specifically during the formation of NETs [29,30]. Therefore, neutrophils typically attempt to sequester iron via lactoferrin [31], host cell lysis or specific inflammatory signaling may paradoxically increase the availability of growth-limiting nutrients. This shift in the microenvironment likely fuels the ‘survivor zoospores’, which might be enabling them to proliferate more aggressively in the presence of immune cells than in their absence. Notably, *Pythium* growth is highly dependent on iron bioavailability [32], which might be another factor supporting *Pythium*. The iron levels in neutrophils against *P. insidiosum* infection should be further demonstrated.

Additionally, the diminished fungicidal capacity in β-thalassemic neutrophils appears fundamentally linked to defects in NET formation. While both groups showed minimal baseline NET production, stimulation with PMA or *P. insidiosum* zoospores induced a robust NETotic response only in healthy neutrophils. Patients with β-thalassemia failed to mount a comparable response, suggesting a significant impairment in NETosis. Given that NETs are critical for immobilizing and neutralizing large extracellular pathogens, these findings suggest that defective NET formation is a primary contributor to the compromised innate immune defense in this population [33,34]. These observations align with previous literature indicating that iron overload, a hallmark of β-thalassemia, has deleterious effects on neutrophil physiology [35]. Additionally, excess iron disrupts intracellular redox balance and impairs the generation of ROS, which serve as central drivers of the NETotic pathway [36]. The generation of ROS in β-thalassemia patients in response to *P. insidiosum* infection should be further investigated. Furthermore, iron toxicity may interfere with key enzymes, such as NE and MPO, while chronic oxidative stress may desensitize signaling pathways to pathogenic stimuli [37,38]. The biological complexity of *P. insidiosum* further underscores the necessity of NET-mediated defenses. As an oomycete capable of forming large zoospores and filamentous hyphae, *P. insidiosum* often evades phagocytosis, requiring extracellular strategies such as NET deployment for containment [12]. Consequently, these results identify neutrophil dysfunction, specifically impaired killing and defective NETosis, as a critical factor in the increased susceptibility of β-thalassemia patients to *P. insidiosum*.

Previous studies suggest that phagocytosis serves as a critical effector mechanism in neutrophil-mediated responses to pathogenic invasion [39,40]. In this study, the phagocytic efficacy of neutrophils against *P. insidiosum* was assessed in β-thalassemia patients and healthy controls. Although neutrophils from both groups were capable of engulfing heat-killed *P. insidiosum* zoospores, a significantly lower phagocytic capacity was observed in β-thalassemia patients compared with healthy individuals. Nevertheless, the overall level of phagocytic activity remained relatively low, suggesting that neutrophils are unable to efficiently sequester *P. insidiosum* zoospores. These findings indicate that phagocytosis is unlikely to represent the primary mechanism responsible for the clearance of this organism. As reported by Sriwarom et al. and Branzk et al., according to the size-dependent hypothesis, this inefficiency may be attributable to cellular dimensions [12,41]. Human neutrophils typically measure 10–12 µm in diameter [42], whereas *P. insidiosum* zoospores range from 8–12 µm [43], which is considerably larger than other common pathogens. Consequently, these findings suggest that neutrophil defense strategies are influenced by microbial size, with a preferential shift toward NET formation when encountering larger pathogens such as *P. insidiosum*. Furthermore, the significantly reduced phagocytic capacity observed in patients with β-thalassemia may reflect underlying neutrophil dysfunction. This interpretation is supported by previous reports demonstrating increased susceptibility to microbial colonization and infection in patients with thalassemia, including significantly higher oral *Candida albicans* colonization in individuals with thalassemia major compared with healthy controls [13].

Hence, the efficacy of this response is severely diminished in patients with β-thalassemia might be associated with chronic iron overload that generates excessive oxidative stress and leads to cellular dysfunction. This metabolic environment results in a reduced phagocytic capacity and defective NET formation, allowing *P. insidiosum* to evade immune clearance and establish persistent infection. To enhance the generalizability of these findings, subsequent research should also explore broader immunological parameters, including neutrophil-associated gene expression and cytokine responses. An additional limitation of this study is the difference in age distribution between the healthy donor and β-thalassemia groups. Although the difference in age between the groups was not statistically significant (*p = 0.0671*), age may influence neutrophil functions, including fungicidal capacity, phagocytic activity, NETotic capacity, and other effector responses [44,45]. Therefore, the possibility that age-related differences contributed to the observed alterations in neutrophil function cannot be completely excluded. Future studies including larger cohorts with more closely age-matched participants or appropriately adjusted analyses will be important to further distinguish the effects of β-thalassemia-associated immune dysfunction from potential age-related effects. Such insights will be vital for understanding the disease and improving outcomes for patients with β-thalassemia.

## Conclusion

These findings indicate diminished fungicidal capacity, phagocytic activity, and NETotic capacity in β-thalassemia patients, which may compromise extracellular antimicrobial defense against *P. insidiosum*. This impairment may partially explain the increased susceptibility to infection and the more severe clinical outcomes observed in patients with pythiosis.

## Supporting information

S1 File Raw data 1. (XLSX)

S2 File Raw data 2. (XLSX)

## References

[1] Rodriguez A, Suo X, Liu D. Chapter 147 - Classification of medically important parasites. In: Tang Y-W, Hindiyeh MY, Liu D, Sails A, Spearman P, Zhang J-R. Molecular Medical Microbiology. Elsevier; 2024. p. 2907–19. doi: 10.1016/b978-0-12-818619-0.00118-0

[2] Zoran DL. CHAPTER 33 - Diseases of the Small Intestines. In: Morgan RV. Handbook of Small Animal Practice. Elsevier; 2008. p. 357–82. doi: 10.1016/b978-1-4160-3949-5.50037-6

[3] Krajaejun T, Sathapatayavongs B, Pracharktam R, Nitiyanant P, Leelachaikul P, Wanachiwanawin W, et al. Clinical and epidemiological analyses of human pythiosis in Thailand. Clin Infect Dis. 2006;43(5):569–76. doi: 10.1086/506353 16886148

[4] Vanittanakom N, Szekely J, Khanthawong S, Sawutdeechaikul P, Vanittanakom P, Fisher MC. Molecular detection of Pythium insidiosum from soil in Thai agricultural areas. Int J Med Microbiol. 2014;304(3–4):321–6. doi: 10.1016/j.ijmm.2013.11.016 24444720

[5] Yolanda H, Krajaejun T. Global distribution and clinical features of pythiosis in humans and animals. J Fungi (Basel). 2022;8(2):182. doi: 10.3390/jof8020182 35205934 PMC8879638

[6] Keoprasom N, Chularojanamontri L, Chayakulkeeree M, Chaiprasert A, Wanachiwanawin W, Ruangsetakit C. Vascular pythiosis in a thalassemic patient presenting as bilateral leg ulcers. Med Mycol Case Rep. 2012;2:25–8. doi: 10.1016/j.mmcr.2012.12.002 24432209 PMC3885954

[7] Reanpang T, Orrapin S, Orrapin S, Arworn S, Kattipatanapong T, Srisuwan T, et al. Vascular pythiosis of the lower extremity in northern Thailand: Ten years’ experience. Int J Low Extrem Wounds. 2015;14(3):245–50. 10.1177/1534734615599652 26286930

[8] Origa R. β-thalassemia. Genetics in Medicine. 2017;19(6):609–19. 10.1038/gim.2016.173 27811859

[9] Naeim F, Nagesh Rao P, Song SX, Grody WW. Disorders of Red Blood Cells—Anemias. In: Naeim F, Nagesh Rao P, Song SX, Grody WW. Atlas of Hematopathology. Academic Press; 2013. p. 675–704.

[10] Walker EM, Walker SM. Effects of iron overload on the immune system. Ann Clin Lab Sci. 2000;30(4):354–65. 11045759

[11] Zhang F, Xia Y, Su J, Quan F, Zhou H, Li Q, et al. Neutrophil diversity and function in health and disease. Signal Transduct Target Ther. 2024;9(1):343. doi: 10.1038/s41392-024-02049-y 39638788 PMC11627463

[12] Sriwarom A, Chiewchengchol D, Saithong S, Worasilchai N, Chindamporn A. Neutrophil extracellular traps and phagocytosis in Pythium insidiosum. PLoS One. 2023;18(1):e0280565. doi: 10.1371/journal.pone.0280565 36693041 PMC9873155

[13] Abd Al Wahab SS, Mahmood MA. Prevalence of Candida albicans in the oral cavity of Beta Thalassemia Major and Thalassemia Minor Patients. F1000Res. 2025;14:555. doi: 10.12688/f1000research.162460.2 40822871 PMC12351238

[14] Billett HH. Hemoglobin and hematocrit. In: Walker HK, Hall WD, Hurst JW. Clinical methods: The history, physical, and laboratory examinations. Boston: Butterworth Publishers, a division of Reed Publishing. 1990. 21250045

[15] Cancado RD, Leite LAC, Muñoz M. Defining Global Thresholds for Serum Ferritin: A Challenging Mission in Establishing the Iron Deficiency Diagnosis in This Era of Striving for Health Equity. Diagnostics (Basel). 2025;15(3):289. doi: 10.3390/diagnostics15030289 39941219 PMC11817370

[16] Mendoza L, Ajello L, McGinnis MR. Infections caused by the oomycetous pathogen Pythium insidiosum. Journal de Mycologie Medicale. 1996;6:151–64.

[17] Gaastra W, Lipman LJA, De Cock AWAM, Exel TK, Pegge RBG, Scheurwater J, et al. Pythium insidiosum: An overview. Vet Microbiol. 2010;146(1–2):1–16. doi: 10.1016/j.vetmic.2010.07.019 20800978

[18] Shintaku T, Glass KA, Hirakawa MP, Longley SJ, Bennett RJ, Bliss JM, et al. Human endothelial cells internalize Candida parapsilosis via N-WASP-mediated endocytosis. Infect Immun. 2013;81(8):2777–87. doi: 10.1128/IAI.00535-13 23690407 PMC3719575

[19] Hellebrekers P, Hietbrink F, Vrisekoop N, Leenen LPH, Koenderman L. Neutrophil Functional Heterogeneity: Identification of Competitive Phagocytosis. Frontiers in Immunology. 2017;8. 10.3389/fimmu.2017.01498 PMC5684128 29170663

[20] Krajaejun T. Pythiosis: An endemic fatal infectious disease. Pathology. 2014;46:S28. doi: 10.1097/01.pat.0000454146.02028.39

[21] Chitasombat MN, Jongkhajornpong P, Lekhanont K, Krajaejun T. Recent update in diagnosis and treatment of human pythiosis. PeerJ. 2020;8:e8555. doi: 10.7717/peerj.8555 32117626 PMC7036273

[22] Gurnani B, Kaur K, Agarwal S, Lalgudi VG, Shekhawat NS, Venugopal A, et al. Pythium insidiosum Keratitis: Past, Present, and Future. Ophthalmol Ther. 2022;11(5):1629–53. doi: 10.1007/s40123-022-00542-7 35788551 PMC9255487

[23] Thongsri Y, Wonglakorn L, Chaiprasert A, Svobodova L, Hamal P, Pakarasang M, et al. Evaluation for the clinical diagnosis of Pythium insidiosum using a single-tube nested PCR. Mycopathologia. 2013;176(5–6):369–76. doi: 10.1007/s11046-013-9695-3 23948967 PMC3843749

[24] Sathapatayavongs B, Leelachaikul P, Prachaktam R, Atichartakarn V, Sriphojanart S, Trairatvorakul P, et al. Human pythiosis associated with thalassemia hemoglobinopathy syndrome. J Infect Dis. 1989;159(2):274–80. doi: 10.1093/infdis/159.2.274 2644370

[25] Laohapensang K, Rutherford RB, Supabandhu J, Vanittanakom N. Vascular pythiosis in a thalassemic patient. Vascular. 2009;17(4):234–8. doi: 10.2310/6670.2008.00073 19698307

[26] Gluba-Brzózka A, Franczyk B, Rysz-Górzyńska M, Rokicki R, Koziarska-Rościszewska M, Rysz J. Pathomechanisms of immunological disturbances in β-thalassemia. Int J Mol Sci. 2021;22(18):9677. doi: 10.3390/ijms22189677 34575839 PMC8469188

[27] Buttari B, Profumo E, Caprari P, Massimi S, Sorrentino F, Maffei L, et al. Phenotypical and functional abnormalities of circulating neutrophils in patients with β-thalassemia. Ann Hematol. 2020;99(10):2265–77. doi: 10.1007/s00277-020-04213-0 32803313

[28] Molloy AL, Winterbourn CC. Release of iron from phagocytosed Escherichia coli and uptake by neutrophil lactoferrin. Blood. 1990;75(4):984–9. doi: 10.1182/blood.v75.4.984.bloodjournal754984 2154270

[29] Frost JN, Wideman SK, Preston AE, Teh MR, Ai Z, Wang L, et al. Plasma iron controls neutrophil production and function. Sci Adv. 2022;8(40):eabq5384. doi: 10.1126/sciadv.abq5384 36197985 PMC9534512

[30] Zhang H, Wu D, Wang Y, Shi Y, Shao Y, Zeng F, et al. Ferritin-mediated neutrophil extracellular traps formation and cytokine storm via macrophage scavenger receptor in sepsis-associated lung injury. Cell Commun Signal. 2024;22(1):97. doi: 10.1186/s12964-023-01440-6 38308264 PMC10837893

[31] Zhao X, Ting S-M, Sun G, Roy-O’Reilly M, Mobley AS, Bautista Garrido J, et al. Beneficial role of neutrophils through function of lactoferrin after intracerebral hemorrhage. Stroke. 2018;49(5):1241–7. doi: 10.1161/STROKEAHA.117.020544 29636422 PMC5915919

[32] Zhu D, Yan L, Sun J, Chen S, Xi Y, Zhang K, et al. Iron overload is associated with increased susceptibility to pulmonary Pythium insidiosum infection in mice. PLoS Negl Trop Dis. 2026;20(4):e0014246. doi: 10.1371/journal.pntd.0014246 42030325 PMC13108862

[33] Grabcanovic-Musija F, Obermayer A, Stoiber W, Krautgartner W-D, Steinbacher P, Winterberg N, et al. Neutrophil extracellular trap (NET) formation characterises stable and exacerbated COPD and correlates with airflow limitation. Respir Res. 2015;16(1):59. doi: 10.1186/s12931-015-0221-7 25994149 PMC4455316

[34] Knopf J, Mahajan A, Muñoz LE, Herrmann M. Formation and Clearance of NETs in Health and Disease. Cells. 2022;11(24):4022. doi: 10.3390/cells11244022 36552786 PMC9776415

[35] Renassia C, Louis S, Cuvellier S, Boussetta N, Deschemin J-C, Borderie D, et al. Neutrophils from hereditary hemochromatosis patients are protected from iron excess and are primed. Blood Adv. 2020;4(16):3853–63. doi: 10.1182/bloodadvances.2020002198 32810223 PMC7448602

[36] Kuźmicka W, Manda-Handzlik A, Mroczek A, Cieloch A, Moskalik A, Demkow U, et al. Iron excess affects release of neutrophil extracellular traps and reactive oxygen species but does not influence other functions of neutrophils. Immunol Cell Biol. 2022;100(2):87–100. doi: 10.1111/imcb.12509 34714958

[37] Kim CH, Leitch HA. Iron overload-induced oxidative stress in myelodysplastic syndromes and its cellular sequelae. Crit Rev Oncol Hematol. 2021;163:103367. doi: 10.1016/j.critrevonc.2021.103367 34058341

[38] Xiao X, Saha P, Yeoh BS, Hipp JA, Singh V, Vijay-Kumar M. Myeloperoxidase deficiency attenuates systemic and dietary iron-induced adverse effects. J Nutr Biochem. 2018;62:28–34. doi: 10.1016/j.jnutbio.2018.08.003 30218980 PMC6263781

[39] DeLeo FR, Allen L-AH. Phagocytosis and neutrophil extracellular traps. Fac Rev. 2020;9:25. doi: 10.12703/r/9-25 33659957 PMC7886055

[40] Uribe-Querol E, Rosales C. Phagocytosis: Our current understanding of a universal biological process. Front Immunol. 2020;11:1066. doi: 10.3389/fimmu.2020.01066 32582172 PMC7280488

[41] Branzk N, Lubojemska A, Hardison SE, Wang Q, Gutierrez MG, Brown GD, et al. Neutrophils sense microbe size and selectively release neutrophil extracellular traps in response to large pathogens. Nat Immunol. 2014;15(11):1017–25. doi: 10.1038/ni.2987 25217981 PMC4236687

[42] Goldsmith LA, Katz SI, Gilchrest BA, Paller AS, Leffell DJ, Wolff K. Regulation of the Production and Activation of Neutrophils. In: Goldsmith LA, Katz SI, Gilchrest BA, Paller AS, Leffell DJ, Wolff K. Fitzpatrick’s Dermatology in General Medicine. 8e ed. New York, NY: The McGraw-Hill Companies; 2012.

[43] Grooters AM, Gee MK. Development of a nested polymerase chain reaction assay for the detection and identification of Pythium insidiosum. J Vet Intern Med. 2002;16(2):147–52. doi: 10.1892/0891-6640(2002)016<0147:doanpc>2.3.co;2 11899029

[44] Shaw AC, Joshi S, Greenwood H, Panda A, Lord JM. Aging of the innate immune system. Curr Opin Immunol. 2010;22(4):507–13. doi: 10.1016/j.coi.2010.05.003 20667703 PMC4034446

[45] Wang Z, Saxena A, Yan W, Uriarte SM, Siqueira R, Li X. The impact of aging on neutrophil functions and the contribution to periodontitis. Int J Oral Sci. 2025;17(1):10. doi: 10.1038/s41368-024-00332-w 39819982 PMC11739572

